# Experiences and actions taken by women to address delayed conception in Delhi, India: A qualitative study

**DOI:** 10.1371/journal.pgph.0006265

**Published:** 2026-05-18

**Authors:** Priyanka Adhikary, Gitau Mburu, Ndema Abu Habib, Rita Kabra, James Kiarie, Neeta Dhabhai, Sarmila Mazumder

**Affiliations:** 1 Society for Applied Studies, New Delhi, India; 2 UNDP-UNFPA-UNICEF-WHO-World Bank Special Programme of Research, Development and Research Training in Human Reproduction (HRP) Department of Sexual and Reproductive Health and Research, World Health Organization, Geneva, Switzerland; The University of Newcastle Australia: University of Newcastle, AUSTRALIA

## Abstract

One in six people experiences infertility in their lifetime globally, including India. Understanding experiences and actions taken by people with delayed conception requires developing person-centred fertility care interventions. We applied the theory of planned behaviour to explore the experiences and actions of women in couples that failed to conceive in low to lower middle-income neighbourhoods of Delhi, India. This was a qualitative study among 35 women who had failed to conceive after 18 months. Data were collected between February and July 2021 through focus group discussions. Deductive thematic analysis was used to identify themes and sub-themes on illustrating experiences and actions taken, guided by the theory of planned behaviour. Attitudes, subjective norms, intentions, actions, and wider contextual factors played a role in influencing whether women took planned actions geared towards their intention of childbearing. These included perceived benefits of having a child, beliefs of mandated motherhood, pro-birth expectations, stigmatised infertility, compliance with family expectations, and perceived loss of control. Specific actions taken by the individuals included: help and treatment seeking from multiple sources (traditional, formal or internet), and engaging in consistent sexual activity. External factors impacted these behaviours, including the availability and cost of fertility care, interruption of healthcare due to COVID-19 epidemic, and lack of psycho-social support from husbands, family, or peers. Fertility intentions alone are not enough to achieve the desired outcome of becoming a mother. Multiple factors at individual, societal, and structural level affect women’s ability to act on their intention of achieving a pregnancy and becoming a mother. Interventions that can modify attitudes, subjective norms, normative beliefs, self-efficacy, and structural interventions to assist women achieve their fertility intentions, include awareness raising on infertility, improving availability and cost of fertility care, and enhancing social and peer support for women experiencing infertility.

## Introduction

Infertility is a disease of the male or female reproductive system defined by the failure to achieve a pregnancy after 12 months or more of regular unprotected sexual intercourse [1]. Globally, approximately one in six people experience infertility in their lifetime [2], resulting in millions of people being affected by it [3]. There is no significant difference in the prevalence of infertility between high-income countries (HIC) and low-middle income countries (LMIC). The lifetime prevalence in HIC is 17.8% and in LMIC is 16.5%, while the period prevalence is HIC is 12.6% and in LMIC is 12.6% [4]. In India, between 3.9% to 16.8% of couples are estimated to have experienced primary infertility [5,6].

A vast amount of research has explored the impact of infertility on people’s lives. Evidence shows that infertility has a negative effect on quality of life, mental health, and well-being [7,8]. Part of these effects are mediated by the way in which infertility is understood in a society, and what it symbolises. In many countries, including India, the ability to bear a biological child is considered an indication of a woman’s health, and her position in the wider family and society [5,9].

Existing research on infertility in India generally falls into epidemiological, biomedical and sociological studies [10–13]. Sociological exploration has resulted in a large body of literature documenting experiences of women with infertility in India [11,14–16]. However, much of these published studies focus on descriptive accounts of infertility experiences and do not document the subsequent actions that women take to cope with infertility or enhance their chances of conceiving [11,14–16]. There is vast literature on stigma, shame, anxiety, depression, mental distress, and intimate partner violence and other psycho-social experiences, but insufficient information on the actions that are prompted by these experiences, when in reality, these are linked [17–22]. However, we posit that research on actions that people take is relevant for several reasons.

First, tracing the link between experiences and actions is an essential first step towards providing them with person-centred fertility care, by shedding light on the determinants of care seeking, and quality of care sought. This is relevant given that some actions can have detrimental effects, while others may be beneficial. Actions taken may involve soliciting assistance from formal biomedical care providers, informal home remedies, folk medicine, religious rituals or homeopathic remedies [23–25]. Some of these actions are ‘irrational and possibly dangerous’ [26] particularly because stigma associated with infertility and social pressures can drive women to take desperate actions to become pregnant while being oblivious to the consequences of such actions [27]. Actions taken can be harmful especially when formal health care is unaffordable, and awareness is low [25,26,28]. Low availability and affordability of fertility care affects health seeking actions as it reduces available options [29]. Because the nature and extent of actions taken by women with infertility can be influenced by individual factors such as information, knowledge [30], and wider social and structural factors [29,31]. Understanding the link between women’s experiences and actions can be leveraged upon to facilitate actions that promote health, while mitigating those that worsen health and well-being.

Second, understanding how experiences prompt a series of actions can assist in uncovering the meanings of these actions. For instance, Maya Unnithan’s research found that treatment seeking behaviours among infertile women in Rajasthan was not just a biomedical pursuit, but carried social meanings [32]. In other words, actions that women take are not only intended to medically resolve a clinical problem, but are also intended to restore a social, psychological, and physical sense of adequacy and completeness [33]. Although help-seeking behaviors are shaped by social and structural factors such as social perception and the availability of health services and how the health sector operates, it is also a cognitive and restorative process that requires our understanding [34].

Third, tracking the link between experiences and the resulting actions can illuminate decision making processes related to care seeking, particularly how their intentions are actualised through specific actions and behaviours. Using theories such as the theory of planned behaviour for example, it is possible to explain the intention and behavior related to seeking a pregnancy [35]. The key tenet in the theory of planned behaviour is that individual actions are primarily driven by intentions, which are influenced by three key factors: attitudes, subjective norms, and perceived behavioral control [36]. Given that the translation of intentions to actual behaviours is influenced by many factors, some of which may be amenable to policy or health intervention, it becomes possible to identify how health behaviours can be improved by: improving attitudes towards healthy behaviours, shifting subjective norms or providing support to improve perceived behavior controls [26]. Therefore, this study aims to document the experiences and actions of women who failed to get pregnant in Northern India.

## Methods

### Study design

This was a descriptive qualitative study.

### Research question

The research question explored in this study was “What are the experiences and actions taken among women who experience delay in conception?”

### Study population and recruitment

The population in this study was women of reproductive age (15–49 years) who completed 18 months without getting pregnant. The population was recruited following their enrolment in a randomised clinical preconception study known as the Women and Infants Integrated Interventions for Growth Study (WINGS) study, whose details are published elsewhere [37]. WINGS was conducted among women from low income to lower middle socioeconomic neighborhoods of urban Delhi who were of reproductive age, sexually active, not using contraception, and not lactating [37,38].

We purposely selected a total of 35 women from a pool of 1530 women, who exited WINGS without getting pregnant after 18 months. To be eligible, women had to have participated in the WINGS study and been unsuccessful in achieving pregnancy over a period of 18 months. Women who had achieved pregnancy in the 18 months of followup in WINGS were excluded [38]. Purposive sampling was used to ensure that women who had no living child as well as those with a living child were included. This approach enabled the research team to capture different experiences from women with primary and secondary infertility, for example women who wanted a second child. Participants were approached via telephonic communication and home visits and invited to participate. They were provided with information regarding the qualitative study and those who accepted were given an appointment for an interview. Recruitment of participants continued until saturation of data. Data saturation occurs when the interviews do not yield additional new information [39].

### Study instrument

To collect data we used a focus group discussion (FGD) guide. The FGD guide contained questions related to experiences and actions of participants including: (i) perception and experiences of delayed conception, (ii) struggles in their failed attempts towards achieving pregnancy, (iii) actions and behaviours that were prompted by their experiences, (iv) coping strategies, and (v) help seeking. FGDs are a useful approach for studying social phenomena, including perceptions and perspectives of participants [39]. The FGD guide was tested among six women in the same locality of Delhi where the study was conducted. Based on the results of the pre-testing, the FGD guide was modified to ensure that questions were understood as intended, were suitable for the study context, and remained sensitive to the stigmatised nature of infertility. Pretesting also ensured that the responses were valid.

### Data collection

Four FGD sessions were held consecutively between 26/02/2021 and 26/07/2021: (FGD1; n = 12), 8 (FGD 2; n = 8), 8 (FGD 3; n = 8), and (FGD 4; n = 7). All the FGDs were conducted in the local language, Hindi. The FGDs were conducted outside the homes of the participants, and not in a healthcare centre to protect participants’ privacy, and to provide a friendly and safe atmosphere where the participants felt comfortable to speak without inhibitions. A neutral place within a homestead was identified to conduct the FGDs. Each FGD lasted between 60 and 90 minutes. The seating arrangement was in a circular fashion having each participant seated in equal distance from one another, which allowed everyone to partake in the discussions with equal chance to express and to be heard clearly. Multiple dicta-phones were placed in and around the room to ensure that the recorded conversations were clearly audible. Gestures including facial expressions, body languages, speech expression, and diction were documented in the field notes to assist in capturing the underlying meanings of participants’ expressions. Each FGD session ended with reviewing the topic guide to ensure that all the questions enlisted in the guide were asked. To confirm that each participant’s response was represented unembellished, each was approached to review all the salient points that were documented as discussed in the FGDs.

Because the moderator shared the same nationality, language, and gender with the participants, she was aware that her positionality could possibly have an impact on participants’ way of addressing questions, their behaviour and reactions, and gestures. The moderator was sentient of her situatedness as an insider which facilitated her in gaining rapport and ease communication with the participants. However, she was also conscious that her positionality as a researcher, shaped by education, and socio-economic class could bias the responses from participants, or influence her way of asking questions. To mitigate the likelihood of the FGDs being influenced by her belief systems, personal experiences, and biases, the moderator constantly self-monitored and remained critically self- reflective throughout the FGDs and maintained neutrality. The moderator (female), a social scientist with doctoral degree in anthropology, was unknown to the participants prior to the FGDs being held.

### Informed consent procedures

Before commencement of the FGDs, the lead researcher (PA) read the aim, benefits, and risks of participating in the study to the participants. This was done to ensure that all participants clearly understood the purpose of the study, bearing in mind that some of them had not attended schools, and may have needed clarification. The lead researcher reminded all the participants that their participation was voluntary, and not mandatory, and they had a right to decline their participation or withdraw from the study at any time. Participants were assured that their refusal or withdrawal from participation would not result in any consequences. Participants were also assured that their anonymity was protected by using unique numerical ID as the only means of identification. Participants were requested to provide written consent by signing an informed consent form. Participants who could not sign provided a thumb impression.

### Theoretical approach

**Fig 1:**
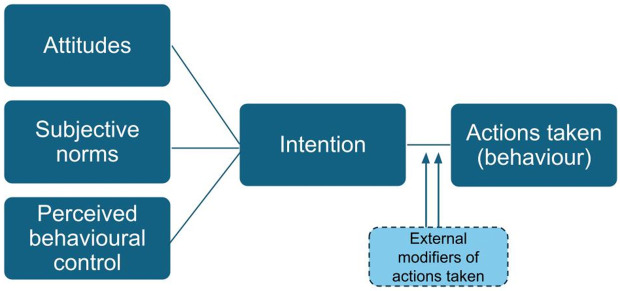
Theory of planned behaviour model, adapted from Ajzen, I. (1991) [36].

The theory of planned behaviour was used to understand the findings of our study. The primary component of the theory is the intention of an individual [36]. Intentions determine to what extent an individual is willing to exert themselves to produce an action and achieve a desired result. A fervent desire for a specific behaviour galvanises the action that is performed. The theory also proposes three essential and corresponding antecedents. Attitude, which refers to an evaluation of whether or not an individual is willing to perform a behaviour is determined by the behavioural belief that lies on subjective notion to occur an event. Subjective norm indicates one’s perception about significant other’s opinion on the performance of a behaviour executed by an individual. Given that behavioural belief stems from personal experiences, the possible relationship between a certain behaviour and attitude could either be affirmative/positive or negative. Subjective norm is swayed by normative belief that reflects the extent to which an individual’s belief system about social expectations coupled with willingness to conform, increase perceived social pressure to get involved in the act. Control belief relates to whether a behaviour needed to fulfill an intention is perceived as *easy or difficult to perform* [40]. It is assumed that a positive attitude strengthens behavioural intention to perform a behaviour. In addition, the theoretical model provides a link to external and contextual factors including those that reflect institutional policies and services, which has been shown to moderate the effect of intentions on goal attainment [41]. Fig 1 titled, *Theory of planned behaviour model, adapted from Ajzen, I. (1991)* shows the graphical representation of the theory of planned behaviour used in this study [36].

### Data analysis

Deductive data analysis was conducted to identify ways in which the data linking the theoretical framework. This deductive analysis was computer-aided, using NVIVO software, version 1.7.1 [42]. To start with, data transcription was performed by the lead researcher and FGD moderator (PA), whose fluency in the local language enabled her to comprehend and interpret participants’ responses and safeguard the merit of the data. All transcripts were then uploaded into the NVIVO software. Transcripts were broken down into small segments and assigned to codes which is a collection of similar ideas, related to the main domains of the theory of behaviour model, i.e., attitudes, subjective norms, and perceived behavioral control, and their antecedents. These codes were populated and then grouped into larger groups which were iteratively labelled to identify emerging sub-themes, while remaining open to discovery, as is the practice in thematic data analysis [39]. Several interlinked sub-themes were then iteratively modified and grouped to create overall themes. The iterative process enabled the assembling of all relevant sub-themes and themes that correspond to the theory of planned behaviour. Emerging codes, sub-themes, and themes were presented to all authors and refined during discussions. Findings were reported in accordance with COREQ guidelines for reporting qualitative studies [43], as shown in supplementary material 1 (S1 Checklist).

### Ethical considerations and approval

The study protocol was reviewed and approved by the ethics committee of Society for Applied Studies (SAS) and the WHO Ethical Review Committee (WHO ERC). Reference numbers (SAS/ERC/RHR-Infertility/2020) and A-ID: A65998 respectively.

## Findings

### Participant characteristics

A total of 35 women were interviewed for the study. Table 1 shows the demographic characteristics of the participants. Participants’ age ranged between 21 and 33 years. Of the 35 women, nine women had a living child. All participants were married and heterosexual; 28 were Hindu, 4 were Muslim, 2 were Christian and one was a Jain. The majority of the participants were homemakers. The average annual household income across the 35 participants was 220,000 Indian Rupees (2674USD, based on prevalent exchange rate at time of writing).

**Table 1:** Participant characteristics.

Social demographic characteristic		Number of participants (N = 35)	Proportion (%)
Age Range	21-25 years	10	28.57%
	26-30 years	14	40%
	30-35 years	11	31.43%
	27.68 years	–	–
Mean age	No formal education	3	8.57%
Education	Elementary school	23	65.71%
	High school	9	25.71
Occupation	Homemaker	33	94.28%
	Small business owner	2	5.71%
Religion	Hindu	28	80%
	Muslim	4	11.42%
	Christian	2	5.71%
	Jain	1	2.85%
Cast	General	12	34.28%
	Scheduled	15	42.85%
	Minority classes	8	22.85%
Parity status	No live birth	26	74.28%
	Achieved live birth	9	25.71%

### Emerging themes

A total of 9 themes and 20 sub-themes emerged from the study related to attitudes (n = 1), normative belief (n = 1), subjective norms (n = 2), perceived behaviour control (n = 2), intentions (n = 1), and actions taken, and determinants of actions taken (n = 2) as displayed in Table 2.

**Table 2:** Emerging themes based on theory of planned behaviour.

Themes	Sub-themes	Codes
**Attitudes towards**		• Child as a source of status, acceptance, and respect.
**having a child**	Perceived benefits of having a child	• Child as a companion.
**Normative belief**	Mandated motherhood	• Normative pressure of gender roles and motherhood • Retaining femininity in failed motherhood and obligations of women to bear a child.
**Subjective norms**	Pro-birth expectations	• Expectation to bear a child within the first year of marriage. • Inquisition and curiosity about delayed conception from family and significant others.
	Motivations to comply with family and societal pressure to have a child	• Desire to meet societal expectations. • Unburdening oneself from the stigma of being childless and regaining respect. • Acquiescing with men’s reluctance to seek help.
**Perceived** **behavioural control**	Loss of control	• Reliance on fate, destiny and God’s will. • Impact of stigma on perceived ability to seek treatment • Impact of costs on perceived ability to seek treatment
	Biological limitations	• Comorbidities as a limitation to perceived control.
**Intention**	Intent for a child	• Desire for own biological child • Consistent sexual activity
**Actions taken**	Help and treatment seeking	• Medical pluralism (doctors, traditional treatments, herbal, faith healers, homemade remedies). • Seeking information from social media and the internet.
	Modifiers of actions taken	• Interruption of healthcare access due to COVID-19. • Lack of psychosocial support from husbands, family and peers.

### Attitudes towards having a child

This theme shows the ways in which attitudes towards having a child, particularly how participants perceived having a child as beneficial.

***Perceived benefits of having a child:*** A child was seen as a source of status, acceptance, and respect. Having a child was positively viewed by all participants. Positive attitudes towards having a child were reinforced and shared with family members and husbands. Having a child was perceived as beneficial that it would help women to achieve the societal privilege that women exercise who proved their womanhood by bearing a child. Many participants felt that having a child would be rewarded with respect and recognition from family and society at large, and their own families would be valued in the eyes of neighbours.

*Becoming a mother would validate and uplift my status in the family and my family would be respected in our neighbourhood,* shared by a 21 year old participant, married when she was 19 years old. (Participant# 2)

A handful of women also indicated that a child provides additional benefits of providing company and companionship. Many shared that they felt lonely, and that having a child could put an end to their loneliness and provide them a sense of joy and purpose in life.

*Once my husband is out for work, I am alone. If I had a child, I could spend time with them. Life seems incomplete without a kid.* (Participant# 27)

### Normative belief

This theme relates to the way in which women’s role as mothers is expected and approved by society in general through a process of social construction, and how such expectations affect women’s behaviour.

***Mandated motherhood:*** Findings showed how normative pressure of gender roles and motherhood played a role in women’s lives. The obligation of motherhood was clearly evident in the voices of the participants. Many participants explained how they carried the responsibility of giving birth on their shoulders.

A 27 year old woman with no children narrated, *since I am married to their son, it is my responsibility to give them a grandchild.* (Participant# 7)

Many women expressed that their life would only be fulfilled, and they would be recognised by society if they had a child within a year after marriage.

*Unless you give birth to a child, you are unrecognised in the society,* said a 29 year old woman with no children. (Participant# 29)

Many of the women accepted their assigned role in the family and society, as explained by a 30 year old participant that never had a child stated that *I have realised over the years that having the identity of a biological mother empowers a woman’s position in society* (Participant# 17). Most of them had seemingly internalised such expectations and perceived motherhood, and linked femininity as indispensable if they were to be respected and accepted in society.

A deep yearning for motherhood reverberated in the voice of a 33 year old childless woman, *I feel I have no other way but becoming pregnant by any means possible. I have been compared with other women of my age who were married and are already mothers.* (Participant# 11)

At the same time, a few women disagreed that they needed to be a mother to be feminine enough. They believed that they would still retain their womanhood even while childless, and their femininity should not be equated with motherhood, which becomes a mandate by marriage:

A 31 year old woman, childless lamented with resentment, *I just wonder sometimes what if I was not born a woman. What if I never had a womb? The onus of carrying a baby and then pushing it out of my body is on me because I am a woman. Now I am struggling to conceive, my femininity is under question?* (Participant# 13)

In support of this assertion, another participant that never conceived was critical of the link between womanhood, and motherhood, which she felt was unfair:

*She is correct. Nobody questions one’s femininity or how complete she is as a woman if she has a kid. It is quite the opposite for a woman who hasn’t been able to be a mother for four years or even eight years. God forbid if somebody remains childless for about 10 years; she is perceived in a certain way. We all know how we are perceived in the eyes of society.* (Participant# 17).

### Subjective norms

This theme illustrates how participants perceived expectations of people around them regarding their becoming mothers, and documents how such subjective norms swayed to think or act in certain ways to comply with these external expectations.

***Pro-birth expectations:*** Findings showed that there was a perceived expectation to bear a child soon after marriage, from family members and neighbours. It was typical to hear participants mentioning that *it is desired to have a baby within a year of marriage* (participant# 7). The expectation to have a child within one year of marriage was emphasised, by other participants who noted that if that expectation was not met, questions arose:

After three years of marriage without offspring, a woman aged 28 expressed with frustration and sadness, *once a couple spends one year of marriage [without getting a child], neighbours start gossiping. Then they start comparing childless couples with those who have a child or children. Then they point out: why are you not getting pregnant. There is a constant pressure on us to have a child.* (Participant# 3)

In several cases, expectations were often turned to questions from family members:

Often compared with female relatives who became mothers before her, tears welled up as the 26 year old woman spoke, *my sister-in-law is married and has kids. I have felt that she tried to raise concerns about my fertility behaviour again and again. Her typical words would be When are you going to have a baby? How long will the medication continue? Why are you not getting pregnant?* (Participant# 14).

Participants were frequently disregarded and disrespected if they did not meet the expectations to have a child, as explained by one participant that her mother-in-law would disdain her state of childlessness and comment, *what good is she for if she cannot bear a child?* (Participant# 7).

Neighbours were also curious about participants’ delayed conception, who also voiced their expectations, often through unwanted questions. Struggling with low confidence and self-esteem because of childlessness and uncertainty regarding the likelihood of conception, a participant shared,

*All that my family wishes for is a child in the family. They told me that no one will dare to raise a finger if I give birth to a child. Some of my neighbours are nosy. Sometimes neighbours can be ten times more annoying than your own family. I try to avoid them whenever they catch a glimpse of me on the street. But they always find a way to ask all sorts of questions that I do not want to discuss.* (Participant# 7)

Multiple accounts showed these expectations created a progressively negative environment because, as communicated by a 25 year old woman that never experienced motherhood,

*Their expectations turned into taunt.*(Participant# 10). Most participants shared that this progressive deterioration occurred as time passed. As a woman of 22 years, having no children disclosed that her situation took a turn for the worse as time went by.

*The situation got worse in my family over a period of time. It started with my mother-in-law who could not control herself anymore from taunting me*. *All this was happening because I was not able to get pregnant.* (Participant# 16)

Failure to achieve motherhood meant that the women were criticised by people known to them, including family members and neighbours. Referring to her family*,* one participant stated that *their expectations turned into taunt,* (Participant# 10), which corroborates the narratives from other participants demonstrating how their family’s unmet expectations created a progressively negative environment.

***Motivation to comply with family and societal pressure to have a child:*** Given these expectations, the desire to meet societal expectations developed among participants. Constant comments coming from families and the society played an important role in persuading participants to take action to comply with the expectations, including seeking medical help.

Family members and their desire for grandchild/children were strong driving forces that swayed participants to acquiesce with the expectations of their significant others:

*My mother-in-law has been persistent that I should go to a godman. I feel compelled to see a godmen as expected by my mother-in-law,* shared by a 23 year old woman who never had a child (Participant# 26)

As demonstrated in the above quote, participants were motivated by their desire to meet expectations from their family, neighbours, and even strangers, who wanted them to validate their existence as a woman by giving birth.

At the same time, women were motivated to comply to unburden themselves from the stigma of being childless and regain respect. Participants sought to regain lost respect from family members, relatives, and neighbours. They desired to be respected in the way that women with children were. To illustrate, a 27 year old childless woman shared,

*Neighbours never raise a concern for a woman who has a child, but they despise childless women.* (Participant# 22)

For a few women seeking help was also acquiescing to men’s reluctance to seek help, given that women were disproportionately blamed when a couple failed to achieve pregnancy, even when their husbands may have been contributing to the failure to get pregnant. With the blame laid on her for being childless and a deep sense of inadequacy, a 27 year old woman despondently said,

*Everybody points at the woman. Nobody realises that it takes both a man and woman to create a life. People behave as if men are born fertile. If something is amiss in terms of reproduction [the fault], must be the woman! As a woman, you are the one who lacks the capability of having a progeny.* (Participant# 31)

Strong conviction among men that women are more likely to be infertile often shaped their perception towards women. A participant explained that her husband was confident that it was not him who had a problem with infertility; rather he claimed it was her who was barren.

Such belief transcended into expectation attributed to subjective norms where some participants self-integrated such belief. These expectations were often reinforced by masculine identity in husbands, as suggested by another participant aged 30, without kids, who, in reference to her husband, said, *his ego is bigger than a mountain. After all, it is about his masculinity which he would never allow anybody to touch.* (Participant# 33). This viewpoint was shared by nearly one third of the participants indicating the fear of emasculation among their husbands as a factor that failed these men to seek help from a doctor, a 30 year old childless participant, married for six years noted that:

*He would not go to any clinic no matter how many times he is told. I know if he did, then his manliness would be put under question. Having said that, become less of a man. He is so firm that the problem lies with me.* (Participant# 8)

Participants perceived men’s virility to be linked with masculine identity. Therefore, to safeguard their identity, men as per the participants, deflected blame onto women which supplemented the common belief that women are responsible for infertility. Participants concomitantly associated such phenomenon with the societal obligation for a baby which dictated their motivation to take some action to fulfil their responsibility conceive a child, as illustrated by a 33 year old woman, married for past eight years, in the following quote:

*I used to feel bad when my husband and mother-in-law impugned me with childlessness, but gradually, I do not really know what happened to me, I started thinking that it is perhaps me who has the problem and I have to fix myself* (Participant#11).

There were, however, a few exceptions where subjective norms were not always experienced by women as a source of societal pressure as highlighted by one participant, who expressed relief for not having to encounter any family pressure, stating that *my family never put me under pressure. God saved me in that way* (Participant# 9). However, for most participants, such expectations and attempts to comply was the norm.

### Perceived behavioural control

In the context of expectations and motivation to comply, this theme demonstrates how the confluence of normative belief and subjective norms influenced participants’ sense of their own ability and efficacy, and prompted them to concretise their intentions and act towards achieving them.

***Loss of control:*** Participants showed varied perceptions of their control over their ability to achieve the desired goal of bearing a child, with many relying on fate, destiny and God’s will. Whereas a few were seemingly in control, the majority came to terms with the fact that they would not be able to achieve their goal, with one 26 year old participant with no children noting that *at the end of it, our fate is not in our control* (Participant# 27). Another participant aged 28 noted how she was in control of the situation after trying for a kid for the past 4 years, revealing that, *we have done our part and still doing* and added that her *husband never says a word about it* (Participant# 6). However, the vast majority had resigned control of the situation. For these women, the outcome was out of their hands, as illustrated by the following excerpt shared by a 31 year old childless woman:

*Maybe bhagwan (God) does not want me to have a kid. Anything and everything will happen when he allows it. I am deprived of his blessings. Maybe I am destined to live my life without a child. Who can defy the will of God?* (Participant# 35)

In addition, findings showed the impact of stigma on perceived ability to act, including seeking treatment. For one participant, physical mobility was compromised as she was restricted to step out of home including seeking treatment or even to spend time with her female friends. The reason described by her was the stigma associated with the state of childlessness of a woman in general compelled her family members to restrict her public movement and her appearance in public spaces. They feared losing dignity within the community.

A 22 year old woman who got married two years ago remarked with a hint of frustration, *my husband’s family members do not like the fact that I go out. They feel that people will start talking about my childlessness. My mother-in-law said that she heard a neighbour labelling me as a barren. They are too worried about our family dignity and image, which they think is dented because of my condition.* (Participant# 13)

Costs associated with seeking treatment also emerged as having an influence on perceived control. Despite the perceived pressure and expectations of family and neighbours, financial obligations restricted women’s ability to fulfil such expectations. Familial expectations were juxtaposed against the cost of treatment, given that the women were financially dependent on their families. This is particularly relevant given that all participants except two were housewives. A participant stated that:

*Sometimes I think that my husband is struggling financially to run the family because of the amount of money we spend on my treatment.* (Participant# 17)

The costs that families either struggled to or were unwilling to spend made them feel that they did not have control on their ability to achieve their goal through treatment. Referring to the family and financial pressures, a participant shared that *money becomes a huge deal for my family members at times and yet I feel the pressure of pregnancy.* (Participant# 15)

***Biological limitations:*** Some women perceived that pre-existing health issues such as thyroid disease, delayed menstruation, diabetes, and polycystic ovaries obstructed their chances of conceiving, and these were largely out of their control. They were already overwhelmed and burdened with the belief that medical conditions were responsible for their inability to conceive. As a 32 year old woman who remained childless despite five years of struggle to conceive a child said,

*It is nothing but my thyroid and diabetes that sabotaged my chances of getting pregnant. At least that is what I believe. But what was more, that, I am outweighed with blame and judgment from others.* (Participant# 4)

### Intention

This theme demonstrates the existence of the extent to intentions related to having a child, which culminates from antecedent attitudes, subjective norms and perceived behaviour control presented previously.

***Intent to have a child:*** All the participants essentially intended to have children and exhibited positive dispositions towards having a child during discussions. Many of them spontaneously expressed their desire for their own biological child, and there was a conscious desire to fulfil that intent:

*I just want to have one baby. My own. Not two or three but only one.* (Participant# 22)

All of them demonstrated fertility intention and were engaged in consistent sexual activity with their partners. However, for some, this was insufficient and further help seeking was necessary.

*My husband and I had regularly engaged in intercourse, and I thought let us just wait if I can get pregnant naturally without any source of help. But now I think the time has come to seek help.* (Participant#16)

### Actions taken

This theme illustrates the steps that participants took in response to their attitudes, subjective norms, normative beliefs and perceived behaviour control. These actions refer to seeking medical help as well as actions that they took to address, manage, and cope with their situation.

***Help and treatment seeking:*** Participants sought help from a variety of sources namely doctors, traditional treatments, herbal, faith healers, and home-made remedies which demonstrated medical pluralism. They attended medical examinations, took medicines, and attended medical procedures such as insemination. These biomedical encounters gave some participants hope. However, for some, seeking biomedical assistance was disrupted by external factors, for instance, status of the health services. Yet, for some seeking support did not yield the results they expected as no treatable cause was found:

*There is nothing wrong with my husband and I. We have both undergone tests which showed that we are perfectly capable of having a child.* (Participant# 9)

For others, doctors’ advice meant that they could no longer take action until their underlying medical issues were resolved. Any effort made without curing the health ailment hindering fertility would be futile, as illustrated by one participant:

*As you see I have thyroid and diabetes, doctors said it will take time for me to get pregnant. I must get myself treated for my thyroid first. Then only I can think of a child.* (Participant# 18)

Some participants sought help from traditional sources, resorting to herbal medicine including *ashwagandha* (Indian Ginseng), *Unani* medicines, or homeopathic remedies. Adopting folk or traditional medicine was reinforced by advice from elderly family members, particularly mothers-in-law, who recommended such medicines to increase fertility. Following the advice of her mother-in-law, a 23 year old participant, married for two years reported as follows:

*My mother-law-law got me Unani medicine from a certain medical store, and I have been advised to take a pill every day at night before I go to sleep.* (Participant# 23)

Similarly, several women reported using home-made remedies, such as nutmeg powder, or milk mixed with turmeric. These were usually consumed daily and for a period such as of two-three months, and often based on advice from family members. One -participant stated*, my mother-in-law always prepares some herbal remedies for me. Sometimes she would offer me a glass of milk mixed in turmeric for a month* (participant # 19). Another reported that, *I used to add nutmeg powder in milk and drink for months once my period gets over. It did not help* (Participant# 14)

Nearly all participants described faith and belief over *baba(s)* (God-men) or god-women or sorcerer as an alternative treatment corresponding to fertility seeking behaviour. A few women kept fasting to please goddess *Sashthi* who, according to the participants, if so pleased can grant a child. The instruction of fasting came from the elderly female members in the family. As illustrated by a participant,

*I have not gone to a doctor per se but my mother-in-law took me to a Jharphook (sorcerer). The way the sorcerer talked to us, it made me feel something is wrong with me that cannot be explained through doctor’s medicine. I am telling you why. While I was sitting in his home, I started feeling dizzy and suddenly my head felt heavy. I almost threw up. I have never had much faith and have always been skeptical about witchcraft and sorcery. My mother-in-law persuaded me to go with her* (Participant# 7)

*Many women in my neighbourhood told my mother-in-law that if I observe fasting dedicated to goddess Sashti who is considered to be the goddess of fertility, I can be bestowed with a child. I do not know if Mother Sashthi will really be pleased and I will be blessed with a child, which I really wish for, I am devotedly praying for a child,* said by a woman who had been keeping fast for the past 3 years for a progeny. (Participant# 24)

For others, neighbours asked them to go and seek help from palm-readers as they believed palm lines influence one’s luck and it’s likely to influence the fate of a childless woman for not having a child of her own. Overall, participants seemed ready to undertake any task or recommended action, and to go to any length to conceive a child.

Notable heterogeneity in treatment seeking behaviour reflected medical pluralism. It was common encounter for participants who believed in several solutions including god-men or *Baba(s)*, occultism or *Tantra* alongside modern medical treatments as corroborated by the following verbatim from a participant:

*I am not relying on dawa (medication) alone, but I also have strong conviction on dua (prayer), and I am praying and humbly asking god for a child* (Participant# 13)

Seeking information from social media and the internet was also a common action that women took. Participants talked about browsing the internet that supposedly guided them towards the possibility of having a child. According to them, they watched videos on YouTube pertaining to the ways of increasing fertility by a doctor who they called *Ladies* doctor. They referred to obstetricians or gynecologists specialised in female reproductive health as the lady doctor. They followed the instructions given by the physician in the video.

I watch YouTube a lot. I regularly follow ******’s videos who talks about herbal medicine that helps getting pregnant and I am persistent in consuming those herbal medicines shown in the videos. (Participant# 26)

***Influence of external factors on actions and behaviours:*** Despite seeking treatment, many contextual barriers were encountered by the participants. For instance, the outbreak of COVID-19 and the subsequent lockdown aggravated the economic crisis for nearly all participants, as the precarious situation made it impossible for them to continue with treatment as some health services were suspended.

*COVID has already caused huge financial loss to my family. On top of that, I spent the utmost that I could for this treatment and that resulted in exhausting all my savings. Now I feel it was all in vain. The money that I used on these medical checkups has just gone to waste. I went to private hospitals as well as private clinics and public healthcare facilities. Now at this point, I am left with no choice but to give up.* (Participant# 21)

In addition, sudden loss of employment during COVID-19 pandemic also affected their will power for a child. For example, one participant shared that:

*My husband lost his job during the COVID lockdown. Acting upon doctor’s order I had gone through IUI right before the lockdown was announced. This procedure did not work, and I did not get pregnant. Now we are penniless.* (Participant# 33)

While a majority stated that their ongoing treatment was hampered due to COVID-19 lockdown and associated job loss, the risk of exposure also scared some of the participants who were reluctant to visit health professionals for seeking medical care, even when lockdowns were lifted. At this juncture, the discussion with the participants suggested that several opted private over public healthcare facilities due to the fear of infection.

While a group of participants preferred private over public healthcare facilities due to fear of catching COVID-19 another group of participants avoided public facilities based on their belief that o*ne cannot get desired treatments in public hospitals* (Participant# 6). Private facilities were preferred as they were less crowded, which saved time:

*Infrastructure in public hospitals is poor. Moreover, everything is time consuming from standing in the queue for your turn to come to see the doctor to getting medicine in the hospital pharmacy. If someone wants to visit a doctor in a public facility, they need to reach by 5 am and then only they can get the prescription. This is no good either. Once the prescription is received then you have to wait for the doctor to come to his/her room which means your entire day is wasted at the hospital just for one checkup.* (Participant# 4)

Lack of psychosocial support from husbands, family, and peers was also an important influence on actions that women implemented. Participants’ accounts gave a notion that they desired for and needed support of their husbands and family members including in-laws, yet the accounts of many indicated that their husbands and family members did not accompany them to clinics. Even female members of their families did not accompany them; they visited clinics alone. Participants’ narratives painted an image of women who were deserted and lonely in the process of resolving their childlessness. A 24 year old woman shared,

*Ironically when it comes to partaking in escorting me to the hospital or a clinic, I see none of the family members are ready to take me* (Participant# 25)

Thus, those who criticised the participants for their inability to procreate were not always ready to lend support in their efforts. In some cases, even husbands backed away from taking their wives to health clinics, leaving the women with a loss of emotional support from people close to them. Speaking of her husband, a participant stated that:

*He would not go to any clinic no matter how many times he is told. I know if he did, then his manliness would be put under question. He is so firm that the problem lies with me.* (Participant# 7)

The fact that emerges in this context is that while families are concerned with a woman’s inability to achieve pregnancy, the responsibility to do so was often left to the woman, with limited social and family emotional support.

## Discussion

This study set out to document the experiences and actions of women who unsuccessfully tried to achieve pregnancy over an 18-month period in Delhi, India. We applied the theory of planned behaviour to explore and elaborate the actions and behaviour of women seeking to have a child. Nearly all the participants demonstrated that they took actions at some point to strengthen chances of achieving their motherhood intentions. While they all had the intention to bear a child, intentions alone were not enough to achieve the desired outcome. Consistent with the theory, our results show the impact of attitudes, subjective norms, and perceived behavioural control on the ability of individuals to achieve their desired goal and intention of having a child. In addition, our study shows that besides attitudes, subjective norms, and intentions, wider contextual factors (including those related to health systems and social support systems) also play a role in influencing whether individuals take actions geared towards the achievement of their goal of childbearing.

Attitudes that influenced these planned actions included perceived benefits of having a child. Results from an international analysis showed that the desire for a child is universally associated with starting families, in contrast to other personal or social factors that tend to be context specific [44]. In contrast to our study, studies examining the impact of attitudes have explored its effect on future rather than present intention, but have nevertheless shown that positive attitudes (e.g., possibility of having a child can provide companionship and can increase joy and satisfaction) enhances such intention [45].

Normative beliefs included beliefs of mandated motherhood, while subjective norms included pro-birth expectations, and normative family expectations. Our finding that women in our study had accepted the idea of mandated motherhood through a socialisation process that emphasised childbearing as being central to women’s value in society has been demonstrated in other sociological studies [14,33]. Mandated motherhood intersected with pro-birth expectations to create a negative environment that had progressively worsened because of participants’ unfulfilled fertility goals over a period of 18 months. It is not surprising that participants’ actions and their actions highly reflected conformity to subjective norms and societal expectations of motherhood. In this context, conventional gender roles were internalised by the participants, and since women’s identity depended on their performance, perceived pressure acted as a strong catalyst influencing mothering intention.

Perceived behaviour control identified, demonstrated perceived self-efficacy or loss of control among participants. Our findings showcase that participants felt helpless, and is consistent with general studies among people with infertility who tend to be resigned to fate, despair, and helplessness as reported in many countries [46,47]. At the same time, it implies that help seeking could be low among these participants. Studies have shown that internal health locus of control is associated with lower odds of help seeking [48]. A perception that the situation is beyond one’s control can further reinforce feelings of self-blame, helplessness, and distress [49]. Furthermore, our study illustrates the important role that physical comorbidities affect the perceived control of participants over their situation. Although physical health has been identified both as a perceived precondition [44,50] and a perceived explanation [46] for achieving or not achieving pregnancy, our findings suggest that it caused participants to perceive less control over their situation.

Actions that participants had undertaken included help and treatment seeking from multiple sources (traditional, formal or internet), as well as adopting sexual intercourse behaviours intended to maximise the chances of conceiving. Our study outcomes correspond with others showing the multiplicity of help seeking from other countries such as Morocco [51]. Being aligned with our study, help seeking to rectify medical or biological problems has also been identified as an important motivational factor to achieve such goals [44,46]. However, some of these help seeking actions taken may not necessarily be beneficial. In our study complementary and alternative remedies were taken as an adjunct, rather than a replacement for doctors, yet participants reported getting sick after taking some remedies. Other studies from both low and high income settings have also reported that women relied on alternative traditional or folk therapies [26,52–54] or took them while also consulting doctors [51]. Although traditional and folk medicines are often used because they are cheaper compared to modern medicine, studies also suggest that desperation and external pressures can persuade women to use treatment that may lack scientific merit, especially when women did not have adequate knowledge on infertility [30,51,55].

External factors influenced participants’ actions. The ability of participants to act on perceived pressure was negatively influenced by economic costs of treatment seeking, access to healthcare, and availability of support from those around them. The COVID-19 lockdown meant that fertility services were no longer available, and women could not access them, and at the same time fear of catching infection was prominent among our participants. Studies conducted in other countries also found that COVID-19 hindered treatment due to the closure of fertility clinics or fear of infection [56,57]. In our study, the demand for fertility care was driven by the wider economic impact posed by COVID-19. For example, job losses of male spouses jeopardised their treatment seeking behaviour. These findings are consistent with earlier reports from our study setting reported by Adhikary et al [58] showing that women’s experiences of infertility are shaped by intersection of gender, economics and masculinity factors that do not act in isolation but are interlinked.

Notably heterogeneity in experiences was also identified, for instance some participants did not experience significant social pressures. In addition, our study supports the idea that the influence of attitudes, subjective norms, and perceived control could differ between those with a child and those who do not have a living child [59]. We observed that women that already had one child seemed to be cushioned from social pressures compared to those without any child. They were less stigmatised since they had already proved their ability to become mothers. On the other hand, women that never had relatively worse experiences including losing a sense of self, feelings of sadness, and loneliness. The isolation and loneliness accentuated the perceived benefits of a child as a companion. Similar findings were documented in previous studies where emotional distress was higher among women with primary infertility compared to secondary infertility [60–62].

### Implications for research health and social policy and interventions

Our results suggest the need to address contextual factors that affect the achievement of childbearing goals, as these can moderate the translation of intentions to behavioural actions or influencing intentions by affecting perceived control. Health systems factors (congestion, user fees) had an impact on the way that intentions were actioned, implying that reducing the costs of treatment and waiting times at public facilities can support achievement of participants’ intentions. In addition, our study suggests that there is a need for family and peer support to assist women navigate and cope with the experiences of treatment seeking. Other studies have also shown the importance of the health care infrastructure, social support policies, [50,63] and potential costs [44,63] in supporting plans to have a child. Furthermore, the presence of social support has been shown to be important in the initial decision on whether to have a child or not [35,64] and while our population already decided they would have a child. The prominence of this concept of social support emphasises its importance even during the treatment seeking journey.

Overall, there is also a need to enhance information and education on infertility in our setting. A rampant lack of information on infertility exists in many countries, and researchers have identified the need for infertility information to enhance perceived control [48], destigmatise infertility [65], and assist in help seeking [66]. In our study setting; information, education, and communication related to infertility can be provided through information campaigns in basic health care facilities. In the communities, through health extension workers focusing on enhancing awareness about infertility, combating stigma, informing communities about the potential danger of some types of traditional therapies, and transforming the gendered perception of infertility as a women’s responsibility.

### Implication for future research

Our findings advance the application of the theory of planned behaviour in studies of infertility. Although the theory has been used in fertility-related studies, it is more commonly used to understand individual intentions to have or limit childbearing among presumed fertile individuals [41]. It is rarely used to gain insight on the intent to have children among people with infertility, or those intending to have a child, but experiencing delay in conception. By focusing exclusively on non-contraceptive women who intended to achieve a pregnancy this study extends our understanding of the factors that are responsible for the realization or frustration of pregnancy intentions, using theory of planned behaviour.

Future studies could adopt some of the attitudes, norms, and control constructs identified in this study. Other researchers have noted that, given that most applications of the theory of planned behaviour model is quantitative [67], typical studies of fertility use presumed attitudes and beliefs [68], rather than those identified from empirical research with representative samples from the target communities [41]. Thus, as argued by other scholars, future quantitative surveys should incorporate ideas and beliefs extracted from real samples [35], noting that beliefs and attitudes vary between communities and over time [35]. The goal of having a child involves specific actions taking place within a specific context, over a specific period, and therefore contextualising fertility research is critical.

In terms of the application of the theory, an important issue to note is that, although having a child, is commonly described as fertility behavior, we considered it as an outcome of one or more antecedent behaviors (e.g., having regular unprotected heterosexual intercourse, and not using a contraceptive). Thus, in our application of this theory, achieving pregnancy is used as a proxy indicator of the results of other actions and behaviours geared toward this outcome. Our study demonstrates that although women may have greater control of some antecedent behaviours, they have less control over the desired outcome. Thus, unprotected sex, the behavior, could be intentional, even if the desired outcome, i.e., pregnancy, was not intended [69].

### Limitations

Our study is limited by the nature of the sample, only married people, who intended to conceive but were unable to do so after 18 months were recruited into the study, which means other perspectives (e.g., from single women) were not captured. Our study did not include men, yet perspectives from men are important, given that the decision making and the influence of attitudes (such as desire for a child), subjective norms, and other antecedent factors related to intentions and decision making to have a child could differ between by gender [36].

## Conclusion

This study shows that fertility intentions alone are not enough to achieve the desired outcome of becoming a mother. Attitudes, subjective norms, and behaviour control affect the ability of women to act on their intention of achieving pregnancy and attaining their goal of having a child. The wider context, including health services and affordability and the wider socioeconomic and cultural environment, also affect women’s actions to actualise their goal. These findings show the need for interventions that can modify attitudes, subjective norms, normative beliefs, and self-efficacy as well as structural interventions to assist women achieve their fertility intentions, including awareness raising on infertility, improving availability and cost of fertility care, and enhancing social and peer support for women experiencing infertility.

## Supporting information

S1 Checklist COREQ Checklist. (DOC)
